# Comparative biotoxicity study for identifying better alternative insecticide especially green nano-emulsion which used as mosquitocides

**DOI:** 10.1186/s12917-024-03992-2

**Published:** 2024-04-20

**Authors:** Muhammad S. M. Shamseldean, Marwa M. Attia, Reda M. S. Korany, Nehal A. Othman, Sally F. M. Allam

**Affiliations:** 1 Applied Center for Entomonematodes, Department of Zoology and Agricultural Nematology, Faculty of Agriculture, Cairo University, Giza, 12613 Egypt; 2 Department of Parasitology, Faculty of Veterinary Medicine, Cairo University, Giza, 12211 Egypt; 3 Department of Pathology, Faculty of Veterinary Medicine, Cairo University, Giza, 12211 Egypt

**Keywords:** Chitosan nanoparticles, Chitosan-silver nanocomposites, Green nanoemulsion, Mosquitocides

## Abstract

This research work was planned to test biosafety of different nanomaterials on the different animals models. These nanoparticles were previously used as potential insecticides of mosquito larvae. The biosafety of these nanoproducts were evaluated on certain organs of non target animals that associated with mosquito breeding sites in Egypt. Animal organs such as the kidneys of rats, toads, and the fish’s spleen were used as models to study the biological toxicity of these nanomaterials. After 30 days of the animals receiving the nanomaterials in their water supply, different cell mediated immune cells were assessed in these tissues. Both TNF-α and BAX immuno-expression were also used as immunohistochemical markers. Histopathology was conducted to detect the effect of the tested nanoproducts at the tissue level of the liver and kidneys of both the rats and toads. Green nanoemulsion of the lavender essential oil was relatively more effective, safe, and biodegradable to be used as insecticides against mosquito larvae than the metal-based nanomaterials.

## Introduction

Mosquitoes are widespread insect pests that are members of the family Culicidae (Order: Diptera). Both humans and animals can contract dangerous diseases from these insect pests such as malaria and different arboviruses such as West Nile Fever virus; St. Louis encephalitis, and Japanese encephalitis viruses. According to the [1, 2], mosquitoes are the deadliest vectors of diseases in the entire world, killing millions of people annually. Synthetic chemical insecticides such as chlorinated hydrocarbons, and organophosphates which remain the most used agent for mosquito invasion, especially in the third world countries. There is now a pressing need for safe bio-pesticides such as botanical pesticides to replace these detrimental chemicals [3, 4].

It is well known now that synthetic chemical pesticides are harmful to the ecosystems, human and animal health, as well as other beneficial living organisms [5]. Recently, there has been an upsurge interest in herbal pesticides, particularly plant essential oils [6, 7]. Different chemical and/or natural pesticides were employed to control various mosquito different life stages, however research has shown that some of these pesticides are damaging the environment and hurting humans, plants, and/or animals. The majority of these control agents have also encountered varying degrees of resistance from mosquitoes, and they nearly lost their effectiveness as potent mosquito killers [8, 9].

The major issue with employing essential oils in natural settings is their extreme volatility and ensuing instability. To address this issue, nano formulations should be applied. They usually improve the stability and effectiveness of any drug [10–12]. Nanoemulsion formulations result in greater adsorption and less material consumption because of the tiny droplet size, transparency, and long-term physical stability without producing any apparent coagulation, precipitation, and/or biphasic particles [13, 14]. Nano encapsulation prevents chemical reaction with oxygen, moisture, or light; additionally, they have lesser side effects, longer shelf life, and regulates the release of the active ingredients [15, 16]. Moreover, several nanoparticles derived from plant such as chitosan nanocapsules of tarragon (*Artemisia dracunculus*) essential oil and other plant-derived nanoparticles were potent against mosquito larvae with low cytotoxicity [17–19].

The creation of novel, effective, and environmentally safe mosquito control products is now urgently needed [20]. These products should be biodegradable [21] and potent against the target insect pests [22]. According to Murugan et al. [23]. and Benelli et al. [24]. plants produce bioactive organic chemicals that can serve as food deterrents, repellents, and/or growth inhibitors. Thus, in the present work nanomaterials were screened as safe and biodegradable materials used to control mosquito larvae by testing their impact on certain organs of animals coexist in or near the habitats of the aquatic immature mosquito stages, such as fishes, toads and rats.

## Materials and methods

### Safety of the tested nano materials on the rat kidneys; toad and the fish’s spleen

Fishes, toads, and rats are animals live in and/or near water canal infested with mosquitoes in Egypt. They were used as animal models to evaluate the safety of the used nanomaterials as biodegradable and safe insecticides against mosquitoes. The current study was approved by the ethical committee of the Faculty of Veterinary Medicine; Cairo University with the code: VetCU12/10/2021/370.

### The tested chemicals for each group in rats; fish and toad

In this experiment; nanomaterials (chitosan; silver nanoparticles; chitosan- silver nanocomposites; and lavender nanoemulsion) were purchased from Nanotech®^;^ Egypt. The concentrations were selected in accordance with the data recorded by Sadek et al. [25]. .

A. Group I (the control) didn’t get any additives.
B. Group II: chitosan nanoparticles (500 ppm).
C. Group III: chitosan nanoparticles (1000 ppm).
D. Group IV: silver nanoparticles (500 ppm).
E. Group V: silver nanoparticles (1000 ppm).
F. Group VI: chitosan-silver nanocomposite (500 ppm).
G. Group VII: chitosan-silver nanocomposite (1000 ppm).
H. Group VIII: lavender nanoemulsion (500 ppm).
I. Group IX: lavender nanoemulsion (1000 ppm).

**A: The tested rats**: twenty seven adult male Sprague-Dawley rats, weighing between 120 and 180 g each, were procured from Faculty of Veterinary Medicine, Cairo University, Egypt. Before the experiment started, all rats underwent a 2-week acclimatization period. Basal feed and tap water were available to them all at any time. Nine equal groups of rats were randomly assigned (*n* = 3). All of the investigated nanomaterials were administrated orally to the examined rats using stomach tubes; administration over a 30-day period. The rats after 30 days of accommodation and administration of daily nanoparticles; injectable dosage of anesthetic solution S/C; Diazepam (Valium^®^)3–5 mg/kg SC; then followed by cervical dislocation and all organs were dissected and labeled in plastic cups for histopathological and immunohistochemical analysis.

**Determination of serum urea and creatinine level**: serum urea was measured by enzymatic colorimetric urea kit as described by Fawcett and Scott [26].While, serum creatinine was determined with an enzymatic colorimetric creatinine kit according to the method of Schirmeister et al., [27].

**B: The tested African-Egyptian toads**: twenty seven sexually mature males and females of the African-Egyptian toads which were purchased from Faculty of Science; Cairo University; these African toad belongs to the family: Bufonidae; species: *Sclerophrys regularis* (Reuss, 1833) weighing 45–50 g each were used. The toads were maintained in the laboratory at 22 °C and fed earth worms twice a week. They were kept in large glass aquaria with some water that was changed twice daily. Nine equal groups (each group had 3 toad). of toads were formed at random. All the examined nanoparticles were administrated orally for 30 days in the drinking water.

**C: The tested swordtail fishes**: the toxicity tests were conducted on swordtail fish that were three months old which purchased from aquaculture lab in Kafrelshikh Governorate; these fishes had a mean total body weight of 0.39 0.01 g (mean ± SE) and a mean total body length of 2.62 ± 0.05 cm (mean ± SE). Fish were fed pellet feed (TOPMEALTM) at 1% of their body weight prior to the studies in 100 L tanks with a water circulation system and a 16/8 hour light/dark cycle. Fish were moved to separate test vessels with a volume of 10 L each after 7 days of acclimatization, where they were given a further 24 h to adjust before the toxicity trials. Dechlorinated tap water that had been extensively aerated for at least 48 h before to the experiment was utilized in every study. The fish were divided into equal groups at random (*n* = 10). For three days, every nanoparticle concentration was given to a different group of fish.

### Tissue sampling for gene expression analysis in fishes spleen and rats kidneys:

At the end of the experimental period, spleens (indicative organ of the fish health status) and kidneys of the rats were aseptically dissected for gene expression.

**Evaluation of Interleukin 6 and IL-1β activities**: samples from 3 control rats were collected in the same manner used as negative controls.

**RNA isolation**: 100 mg of the rat kidney and fish spleen were used to isolate the mRNA using the total RNA kits (Ambion, Applied Biosystems). Before being put into Lysing Matrix D tubes, the sampled tissues were homogenized using a FastPrep-24 homogenizer (MP Biomedicals, 2 cycles of 30 s at 6 m/s). The purity and quantity of the mRNA were assessed using Thermo Scientific’s Nanodrop. Five-hundred nanograms of mRNA were created using DNase I amplification grade (Invitrogen), as directed by the manufacturer. The reverse transcribed treated mRNA was produced using the High-Capacity cDNA Archive Kit (Applied Biosystems) [28, 29].

**Quantitative real-time PCR protocol** (***q*RT-PCR**): TNF-α; Interleukin-6 and IL-1β PCR primer sets tailored for rats and fishes [31]were created based on sequences stored in the GenBank (Table 1). For sample normalization and as a reference gene, GAPDH was employed. A separate pool of cDNA produced from five healthy rats that had previously been screened for the presence of any parasites was used to evaluate the expression of the genes used in this experiment. Real-time PCR procedure was followed by [30] and Table 2 details their state.

**Table 1:** The sequences of the forward and reverse primer used in the quantitative real-time PCR

Animal Genes	Gene Sequence [5’-3’]	Accession number	Reference
IL-6 (Rats)	F- 5-AGTAGTGAGGAACAAGCCAGAGC	NM_012589	Qing He et al. 2021.
	R- TTGGGTCAGGGGTGGTTATTG		
IL-1β (Rats)	F- GTGGCAATGAGGATGACTT	XM_032902343	Qing He et al. 2021.
	R- TGGGCTTATCATCTTTCAA		
GAPDH (Rats)	F- ACTTTGGTATCGTGGAAGGACTCAT	NM_001009784	Puech et al. 2015.
	R-GTTTTTCTAGACGGCAGGTCAGG		
TNFα-1. (Fishes)	F-GGTTAGTTGAGAAGAAATCACCTGCA	NM_001279533.1	Praveen et al. 2006.
	R-GTCGTCGCTATTCCCGCAGATCA		
IL-1β (Fishes)	F- TGCACTGTCACTGACAGCCAA	DQ061114.1	Heinecke and Buchmann 2013.
	R- ATGTTCAGGTGCACTATGCGG		
β-Actin F (Fishes)	F-CAGCAAGCAGGAGTACGATGAG	XM_003455949.2	Akbari et al. 2017.
	R-TGTGTGGTGTGTGGTTGTTTTG		

**Table 2:** PCR cycling conditions

Steps	Temperature	Time
Initial denaturation	95 ^o^C	10 min
40 cycles		
Denaturation	95 ^o^C	30 s
Annealing	60 ^o^C	30 s
Extension	72 ^o^C	45 s
Final extension	72 ^o^C	10 min

**Histopathological examinations**: after the rat experiment, kidney from rats and kidney and liver tissues from the Egyptian toads were removed from various groups, fixed in 10% buffered formalin, washed, dehydrated, and then embedded in paraffin wax. Sections of the paraffin-embedded blocks were cut at a thickness of 5 microns and stained with hematoxylin and eosin for histological investigation [32]. Stained slides were examined using a light microscope (Olympus BX50, Japan).

**Histopathological lesion scoring**: histopathological changes were scored as, no changes (0), mild (1), moderate (2) and severe (3) changes, the scoring was determined by percentages as follows: <30% changes (mild change), < 30–50% (moderate change) and > 50% (severe changes) [33, 34].

**Immunohistochemistry**: according to the procedures outlined by El-Maksoud et al., [35], an immunohistochemical analysis was carried out. Kidney tissue sections underwent xylene deparaffinization and graded alcohol rehydration. The endogenous peroxidase activity was blocked by the addition of Hydrogen Peroxide Block (Thermo scientific, USA). By pretreating tissue slices with 10 mM citrate and heating them for 10 min in a microwave, antigen retrieval was accomplished. A rabbit monoclonal anti-Bax antibody [E63] at a concentration of 1:250 (ab32503; Abcam, Cambridge, UK) or TNF-α (dilution 1/100, Cell tech Ltd, UK) was incubated on sections for 2 h. Following a PBS rinse, the sections were incubated for 10 min with Goat anti-rabbit IgG H & L (HRP) (ab205718; Abcam, Cambridge, UK). PBS was used once again to rinse the sections. The 3, 3’-diaminobenzidine tetrahydrochloride (DAB, Sigma) was then incubated with the sections. Haematoxylin was used as a counter stain before mounting the slides. PBS was used as a substitute for primary antibodies as negative controls.

**Bax and TNF-α immunostaining evaluation**: the quantitative immuno-reactivity of Bax and TNF-α was evaluated in the sections of rats kidney in each group [36]. Immunoexpression was analyzed in ten microscopical fields per each section under high-power microscope field (x 400). The percentages of positive stained cells (%) was determined by color deconvolution image J 1.52 *p* software (Wayne Rasband, National Institutes of Health, U.S.A).

### Statistical analysis of the genes expression

SPSS computer program was used for the statistical analysis on gene expression analysis using one-way ANOVA.

## Results

### Evaluation of cell mediated immune response in treated fish with 500 ppm concentration

In group B and D, means of IL-1β in the fish spleen has reached 6.6 and 8.4 respectively. When group F were used mean of IL-1β has become 10.0 (Table 3). In contrast, when group H was used, the IL-1β did not increase than the fish normal control of 3.3. When group F were used, means of TNF-α in the fish spleen were 9 and 10.6 respectively. While group F were used mean of TNF-α has become 11.6 (Table 3). While using group H didn’t affect the TNF-α which was 3.3 equal to the control (Table 3). When group B and D, were employed, the means of IL-6 in the fish spleen were 7.4 and 9.8, respectively. IL-6 production increased to 11.2 when group B and D were combined. The amount of IL-6 remained at the fish’s typical control threshold of 2.0 when group H groups were used (Table 3). The use of group H didn’t affect the levels of expression in the IL-1β, TNF-α, and IL-6 genes (Table 3).

**Table 3:** Expression of the genes in fish spleen when treated with 500 ppm of different nano-particles and nano-emulsions of the tested nano materials

Tested nano-materials	IL-β	TNF-α	IL-6
Chitosan	6.6	9	7.4
Silver	8.4	10.6	9.8
Combination (chitosan + silver)	10.0	11.6	11.2
Lavender	3.3	4.3	4.0
Control	3.3	3.3	3.0

Number indicates the elevation of different genes

The IL-1β recorded as 7.3 and 7.73 in the treated rats’ kidneys of group group B and C, respectively. While IL-1β levels increased to 8.47 and 12.23 respectively when group D, concentrations were employed (Table 4). In the meantime, the application of group F and G has increased the mean levels of IL-1β to 8.10 (Table 4). Contrarily, group H and I, at two doses led to a minor increase in IL-1β levels compared to control rats, with respective means of 4.27 and 4.67 respectively (Table 4).

**Table 4:** Interlukin-1β (IL-1β) gene expression in the kidneys of treated rats with different concentrations of nanoparticles and nanoemulsions

Concentration of the tested nano materials in ppm	IL-1β
Group B	7.30
Group C	7.73
Group D	8.47
Group E	12.23
Group F	8.10
Group G	10.87
Group H	4.27
Group I	4.67
Group A	3.97
LSD_0.05_	1.71

Number indicates the elevation of different genes

When group (B and C) were applied means of IL-6 in the rats kidneys were 3.87 and 4.60 respectively. While applying group D and E had used means of IL-6 to reach 8.87 and 11.97 respectively. In contrast, group F; G were 8.43 and 10.03 in F; G respectively. While group H and I were used, the IL-1β slightly elevated above the control rats to reach 4.60 and 5.20 for the two concentrations (Table 5).

**Table 5:** Interlukin-6 (IL-6) gene expression in the kidneys of treated rats with different concentrations of the tested nano-materials

Tested Material	IL-6
Group B	3.87
Group C	4.60
Group D	8.87
Group E	11.97
Group F	8.43
Group G	10.03
Group H	4.60
Group I	5.20
Group A	3.89
LSD_0.05_	0.79

Number indicates the elevation of different genes

**Fig. 1:**
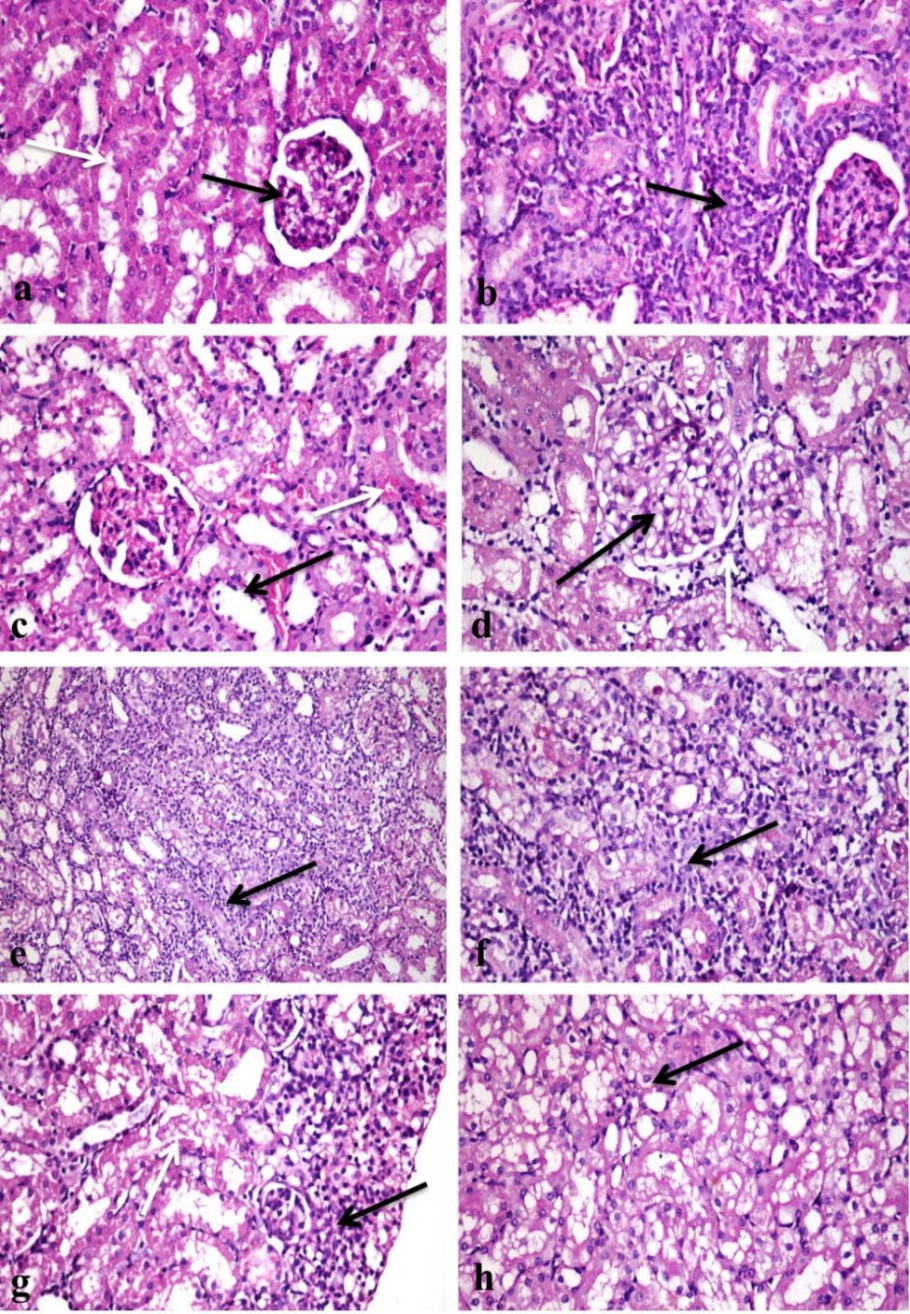
Photomicrographs of sections in kidney tissues of different groups of rats treated with different nanomaterials: (**a**) A section in the control group showing normal histological structure of renal corpuscles (black arrow) and renal tubules (white arrow) (H & E X400). (**b**) A section in the group treated with chitosan nanoparticles B, showing interstitial mononuclear inflammatory cells infiltration (arrow) (H & E X400). (**c**) A section in the group treated with chitosan nanoparticles B, show necrosis of tubular lining epithelium (black arrow) and congestion of inter-tubular blood capillaries (white arrow) (H & E X400). (**d**) A section in the group treated with chitosan nanoparticles C, note vacuolation of endothelial lining of glomerular tuft (black arrow) with interstitial mononuclear inflammatory cells infiltration (white arrow) (H & E X400). (**e**) A section in the group treated with chitosan nanoparticles C, reveal heavy infiltration of interstitial tissue with mononuclear inflammatory cells (arrow) (H & E X200). (**f**) Higher magnification of the previous photo showing interstitial mononuclear inflammatory cells infiltration (arrow) (H & E X400). (**g**) A section in the group treated with chitosan nanoparticles C, show mononuclear inflammatory cells infiltration (black arrow) with renal tubular necrosis (white arrow) (H & E X400). (**h**) A section in the group treated with chitosan nanoparticles C, note vacuolar degeneration and necrosis of tubular lining epithelium (arrow) (H & E X400)

**Fig. 2:**
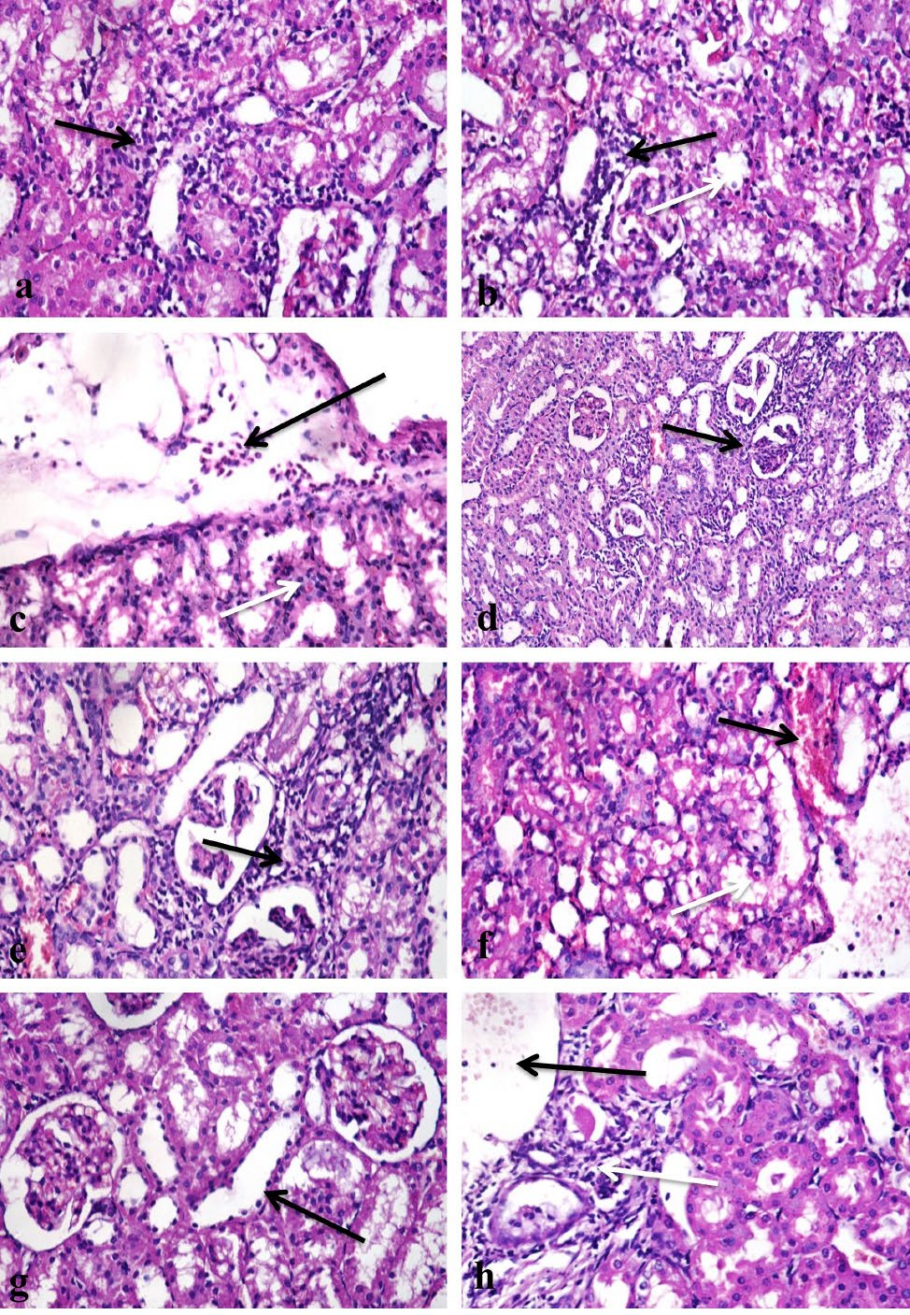
Photomicrographs of sections in kidney tissues of different groups of rats treated with different nanomaterials: (**a**) A section in the group treated with group D, showing few mononuclear inflammatory cells infiltration (arrow) (H & E X400). (**b**) A section in the group treated with group D, note mononuclear inflammatory cells infiltration (arrow) (black arrow) and degeneration of renal tubular epithelium (white arrow) (H & E X400). (**c**) A section in the group treated with group E, showing capsular edema and inflammatory cells infiltration (black arrow) with interstitial mononuclear inflammatory cells infiltration (white arrow) (H & E X400). (**d**) A section in the group treated with group E, note heavy infiltration of interstitial tissue with mononuclear inflammatory cells (arrow) (H & E X200). (**e**) Higher magnification of the previous photo showing mononuclear inflammatory cells infiltration (arrow) (H & E X400). (**f**) A section in the group treated with group E, showing congestion of interstitial blood vessels (black arrows) with vacuolar degeneration of tubular epithelium (white arrows) (H & E X400). (**g**) A section in the group treated with group F showing cystic dilatation and necrosis of renal tubule (arrow) (H & E X400). (**h**) A section in the group treated with group F, showing congestion of interstitial blood vessels (black arrow) and mononuclear inflammatory cells infiltration (white arrow) (H & E X400)

**Fig. 3:**
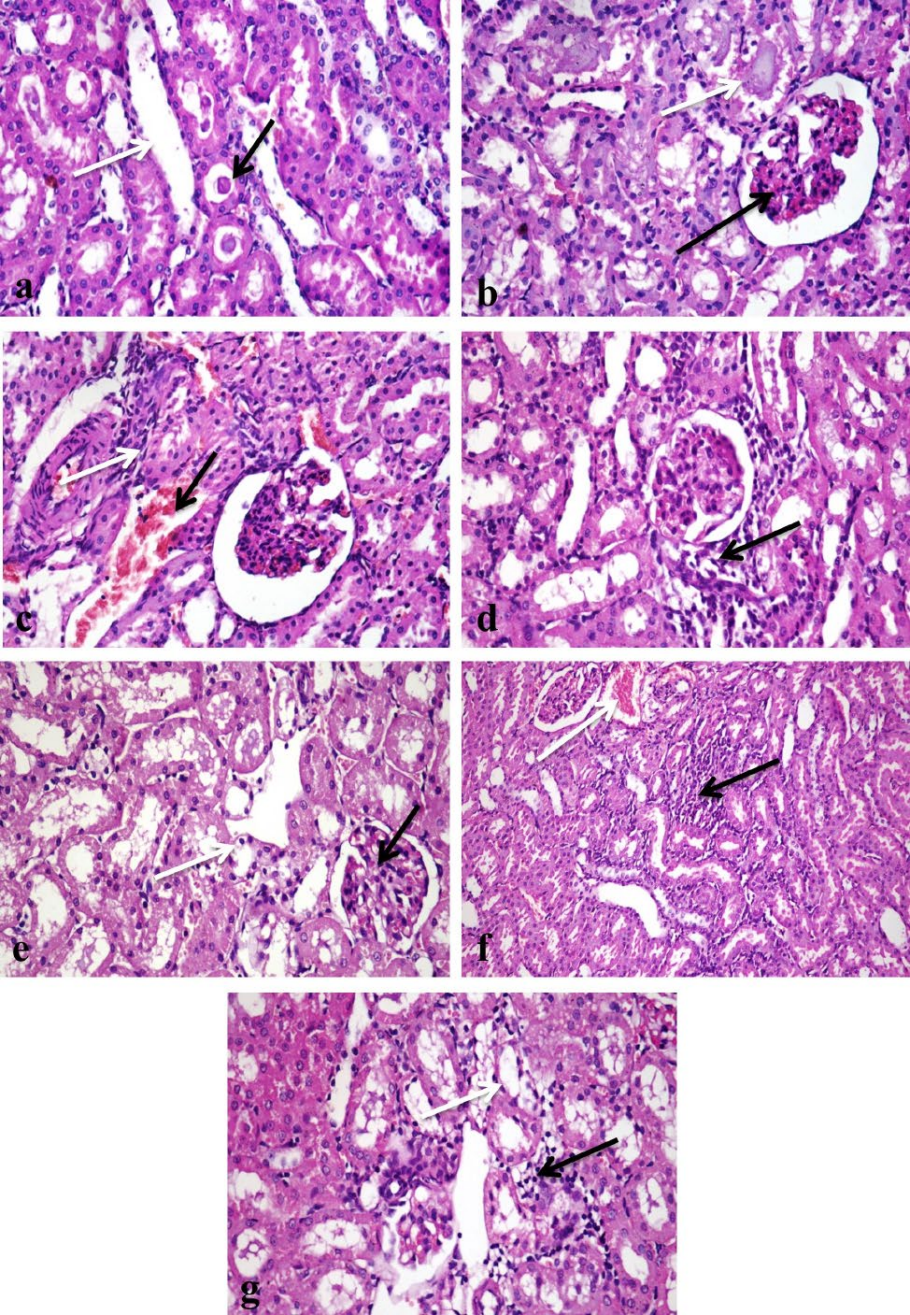
Photomicrographs of sections in kidney tissues of different groups of rats treated with different nanomaterials: (**a**) A section in the group treated with group F, showing tubular cystic dilataion (white arrow) and renal tubular casts (black arrow) (H & E X400). (**b**) A section in the group treated with group G, note glomerular congestion (black arrow) and tubular necrosis and casts (white arrow) (H & E X400). (**c**) A section in the group treated with group G, show congestion of interstitial blood vessel (black arrow) with few mononuclear inflammatory cells infiltration (white arrow) (H & E X400). (**d**) A section in the group treated with group H, showing few mononuclear inflammatory cells infiltration (arrow) (H & E X400). (**e**) A section in the group treated with group H showing mild glomerular congestion (black arrow) and mild tubular epithelial vacuolation (white arrow) (H & E X400). (**f**) A section in the group treated with group I, note focal area of mononuclear inflammatory cells infiltration (arrow) (H & E X200). **g**) A section in the group treated with group I showing few mononuclear inflammatory cells infiltration (black arrow) with mild tubular degeneration (white arrow) (H & E X400)

**Histopathological findings**: Untreated group revealed normal structure of the rat kidneys (Fig. 1a), The rat group treated with group B showed few interstitial mononuclear inflammatory cells infiltration (Fig. 1b) with congestion of glomerular tuft and interstitial blood vessels, few renal tubules showed vacuolation or necrosis of tubular lining epithelium (Fig. 1c). The rat group treated with group B revealed vacuolation and thickening in glomerular tuft (Fig. 1d), heavy infiltration of mononuclear inflammatory cells in interstitial tissue (Fig. 1e and f, and 1g), vacuolation and necrosis were detected in considerable number of tubular lining epithelium (Fig. 1h) with congestion of interstitial blood vessels. The rat Group treated with group D conc. showed few infiltration of interstitial tissue with mononuclear inflammatory cells (Fig. 2a) with vacuolation of tubular lining epithelium and glomerular congestion (Fig. 2b). In contrast, group E showed capsular thickening with edema and inflammatory cells (Fig. 2c), interstitial mononuclear inflammatory cells infiltration (Fig. 2d, and 2e), glomerular and interstitial blood vessels congestion with vacuolation and necrosis of tubular lining epithelium (Fig. 2f**)**. Rat group F revealed cystic dilatation and necrosis of few number of renal tubules (Fig. 2g) with few interstitial mononuclear inflammatory cells infiltration and interstitial blood vessels congestion (Fig. 2h**)**, some renal tubules showed renal casts inside their lumen (Fig. 3a). The group G showed congestion of glomerular tuft and interstitial blood vessels with renal tubular casts (Fig. 3b), there was few mononuclear inflammatory cells infiltration (Fig. 3c) and vacuolation or necrosis in some renal tubular epithelium. Meanwhile, rat groups treated with the two concentration of group H revealed the presence of few mononuclear inflammatory cells infiltration in interstitial tissue with mild interstitial and glomerular congestion (Fig. 3d, and 3f) and vacuolation in few tubular lining epithelium (Fig. 3e, and 3g).

**Histopathological lesion score**: recorded lesions in rat kidneys were scored as shown in Table (6).

**Table 6:** Scoring of histopathological alterations in kidneys of the treated rat groups^*^

Lesions	G. A	G.B	G. C	G. D	G. E	G. F	G. G	G. H	G. I
Congestion of the glomerular tuft.	0	1	2	1	2	1	1	1	1
Congestion of interstitial blood vessels.	0	1	2	1	3	1	2	1	1
Mononuclear inflammatory cells infiltration in interstitial tissue.	0	1	3	1	3	1	2	1	1
Renal tubular vacuolar degeneration.	0	1	3	1	2	1	1	1	1
Renal tubular necrosis.	0	1	2	1	2	1	1	0	0

^*^Each group (*n* = 3), The score system was designed as: score 0 = absence of any lesions in the kidneys; score 1= (< 30%), score 2= (< 30 – 50%), score 3= (> 50%)

**Fig. 4:**
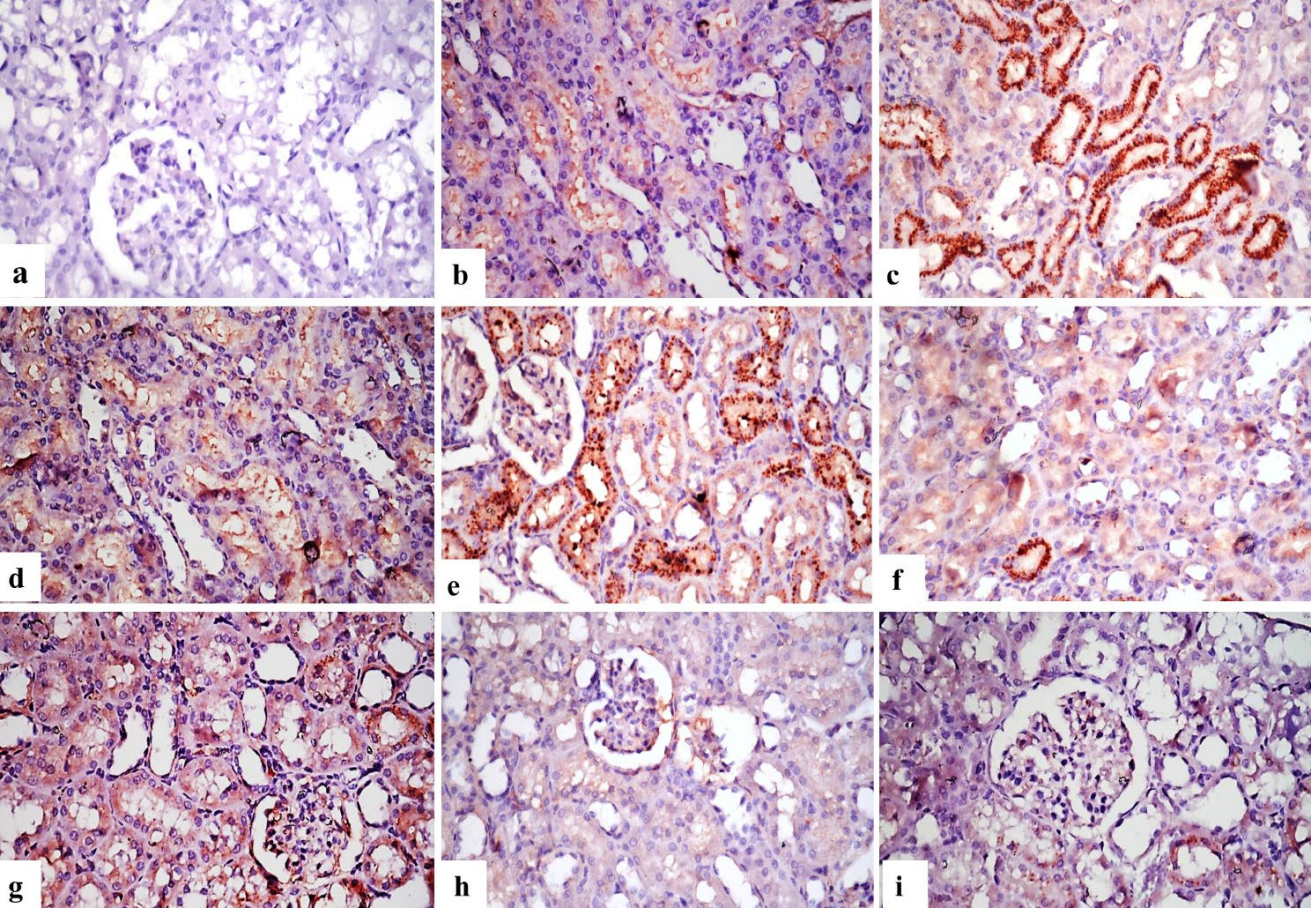
Photomicrographs of sections in kidney tissues of different groups of rats treated with different nanomaterials to demonstrate immunostaining of BAX: (**a**) A section in the control group of rats showing no BAX immune-reactive cells in kidney tissue. (**b**) A section in the group treated with group B, showing weak positive expression of BAX. (**c**) A section in the group treated with group C, showing strong positive immune expression. (**d**) A section in the group treated with group D, show weak positive expression of BAX. (**e**) A section in the group treated with group E, reveal strong positive immune expression. (**f**) A section in the group treated with group E, showing weak expression of BAX. (**g**) A section in the group treated with group G, showing weak expression. (**h**) A section in the group treated with group H, showing very weak immunoreaction of BAX. (**i**) A section in the group treated with group I, showing very weak immune-expression of BAX (X400)

**Fig. 5:**
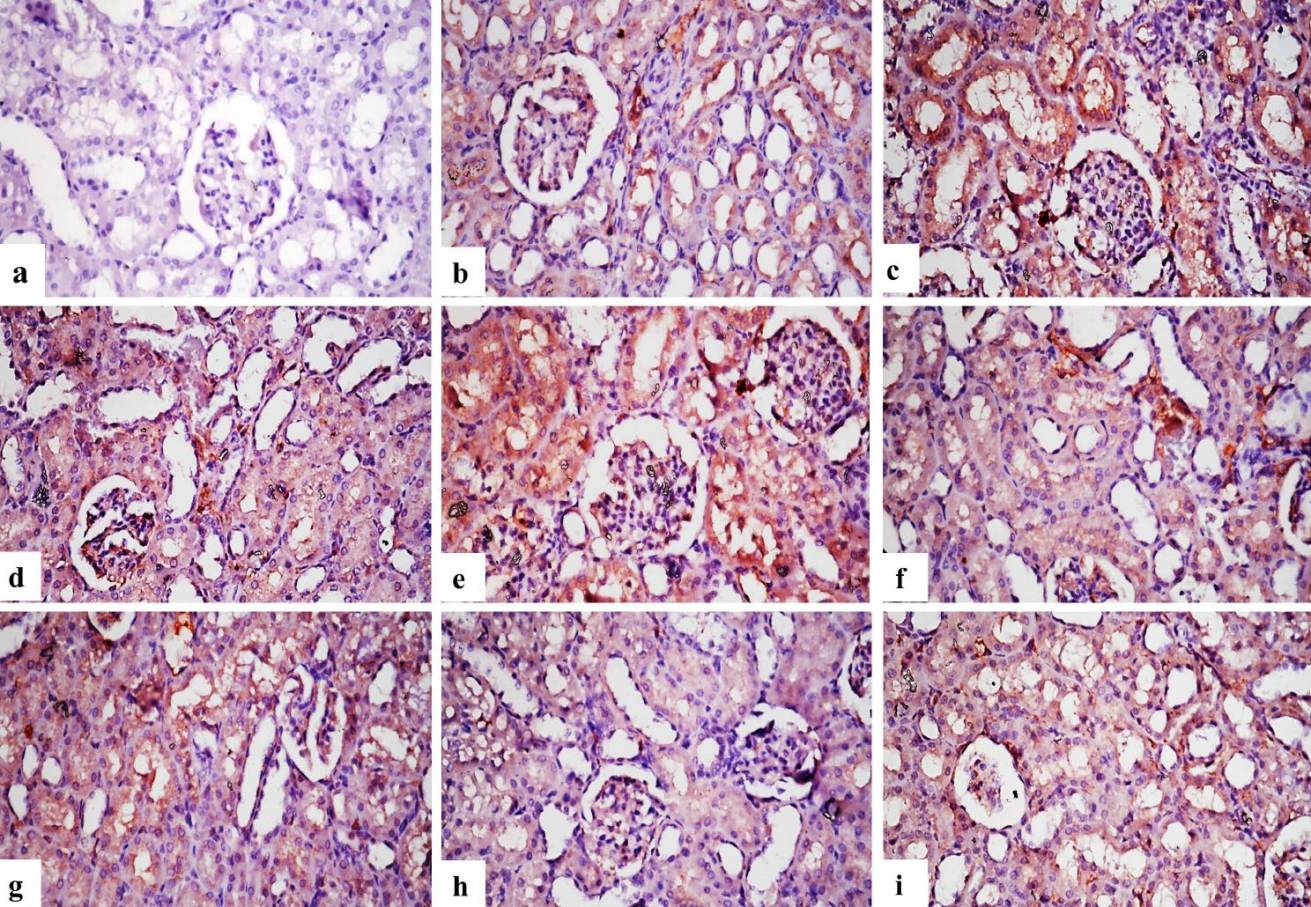
Photomicrographs of sections in kidney tissues of different groups of rats treated with different nanomaterials to demonstrate immunostaining of TNF-α: (**a**) A section in the control rat group showing no TNF-α immune reactive cells in kidney tissues. (**b**) A section in the group treated with group B, showing weak positive expression of TNF-α. (**c**) A section in the group treated with group C, showing strong positive immune expression of TNF-α. (**d**) A section in the group treated with group D, showing weak positive expression of TNF-α. (**e**) A section in the group treated with group E showing strong positive immune expression. (**f**) A section in the group treated with group F, showing weak expression of TNF-α. (**g**) A section in the group treated with group G, showing weak expression of TNF-α. (**h**) Group treated with group H showing very weak immune reaction of TNF-α. (**i**) Group treated with group I, showing very weak immune-expression of TNF-α (X400)

**Immunohistochemical findings for BAX and TNF-α expression**: Table (77) showed the immuno-expression of BAX and TNF**-**α % area in the treated kidneys of different experimental groups. No immuno-reactive cells were found in the control group when BAX and TNF-α immunostaining were applied on the kidney’s tissues (Figs. 4a and 5a). Figure 4b and d, as well as Fig. 5b and d, show that the immunological expression of both markers was poor in the groups that were treated with group B and group D. As opposed to this, BAX and TNF-α indicators were strongly expressed in groups that treated with group C and group E (Figs. 4c and e and 5c and e). Figure 4f, g and h, and 4i, as well as Fig. 5f, g and h, and 5i, indicate immunostaining expression ranging from nil to very faint positive immune-reactions in groups treated with group F; G or group H and I.

**Table 7:** Area % of BAX and TNF-α expression in the kidneys of different experimental groups

	Group B	Group C	Group D	Group E	Group F	Group G	Group H	Group I
BAX	26.7 ± 1.1^b^	56.6 ± 3.9^a^	22.7 ± 0.9^b^	53.3 ± 3.6^a^	15.7 ± 2.5^c^	21.2 ± 2.1^b^	14.5 ± 1.2^c^	15.19 ± 0.6^c^
TNF-α	24.4 ± 1.9^b^	52.8 ± 4.2^a^	22.5 ± 1.2^b^	54.1 ± 6.4^a^	14.9 ± 2.1^c^	23.2 ± 2.9^b^	14.2 ± 1.8^c^	14.9 ± 0.9^c^

Data was expressed as mean ± SE, (*n* = 3). Different letters in the same column were significantly different at (*p* ≤ 0.05)

### Histopathological findings in tissues of the liver and kidneys of the egyptian-african toad

1. Liver Control group showed normal histological structures (Fig. 6a, and 6b), group treated with group D revealed mild vacuolation of hepatocytes with activation of melanomacrophages (Fig. 6c), there was a focal area of mononuclear inflammatory cells infiltration between hepatocytes (Fig. 6d), central vein congestion (Fig. 6e**)** and portal area fibrosis with few mononuclear inflammatory cells infiltration (Fig. 6f). In contrast, group treated with group E, showed massive vacuolation and necrosis of hepatocytes (Fig. 6g), activation of melanomacrophages and central vein congestion, there were portal fibrosis and hyperplasia of bile ducts (Fig. 6h), multifocal areas of inflammatory cells infiltration (Fig. 6i) and infiltration of mononuclear inflammatory cells infiltration in portal areas (Fig. 6j). While the group treated with group B displayed vacuolar hepatocyte degradation along with melanomacrophage activation (Fig. 7a) and central venous congestion. Whereas group treated with group D, revealed vacuolation of hepatocytes with activation of melanomacrophages (Fig. 7b), central vein congestion (Fig. 7c), focal area of inflammatory cells infiltration (Fig. 7d), portal area has fibrosis, mononuclear inflammatory cells infiltration, congestion of portal blood vessels and bile duct hyperplasia (Fig. 7e). In contrast, those who received group F (Ch-Si) exhibited modest hepatocellular deterioration as well as mild central vein congestion (Fig. 7f) and mononuclear infiltration (Fig. 7g). Hepatocytes displayed mitigated central venous congestion and vacuolar degeneration when group G were assessed (Fig. 7h). Isolated areas of sparse mononuclear inflammatory cell infiltration between hepatocytes were also found (Fig. 7i). The group H; I displayed minimal mononuclear infiltration and minor hepatocellular vacuolation (Fig. 7j).
  **Fig. 6:**
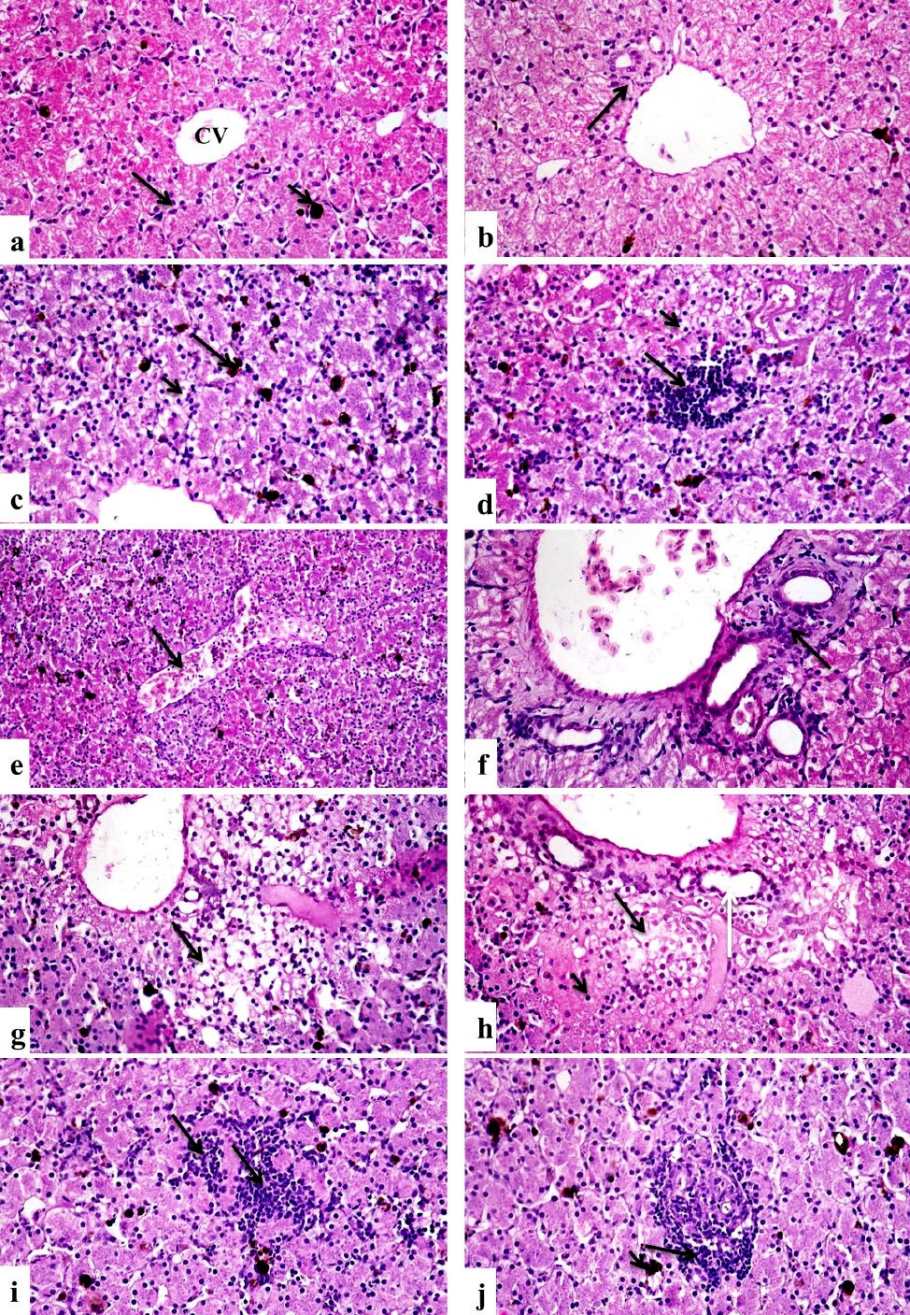
Photomicrographs of sections in the liver tissues of different groups of the African-Egyptian toad treated with different nanomaterials: (**a**) A section in the control group, showing normal histological structure of hepatocytes cord (long arrow), melanomacrophages (short arrow) and central vein (CV). (**b**) A section in the control group showing normal histological structure of portal area (arrow). (**c**) A section in the group treated with group D showing mild vacuolar degeneration of hepatocytes (short arrow) and activation of melano-macrophages (long arrow). (**d**) A section in the group treated with group D, showing focal area of mononuclear inflammatory cells infiltration(long arrow) with hepatocellular vacuolar degeneration (short arrow). (**e**) A section in the group treated with group D, showing central vein congestion (arrow). (**f**) A section in the group treated with group D, showing portal area fibrosis and few mononuclear inflammatory cells infiltration (arrow). (**g**) A section in the group treated with group D, showing massive vacuolation and necrosis of hepatocytes (arrow). (**h**) A section in the group treated with group E, showing vacuolation (long arrow) and necrosis (short arrow) of hepatocytes, portal fibrosis and hyperplasia of bile ducts (white arrow). (**i**) A section in the group treated with group E showing multifocal areas of mononuclear inflammatory cells infiltration (arrows). (**j**) A section in the group treated with group E, showing infiltration of portal areas with mononuclear inflammatory cells infiltration (long arrow) with activation of melano-macrophages (short arrow) (H &E X 400)
  **Fig. 7:**
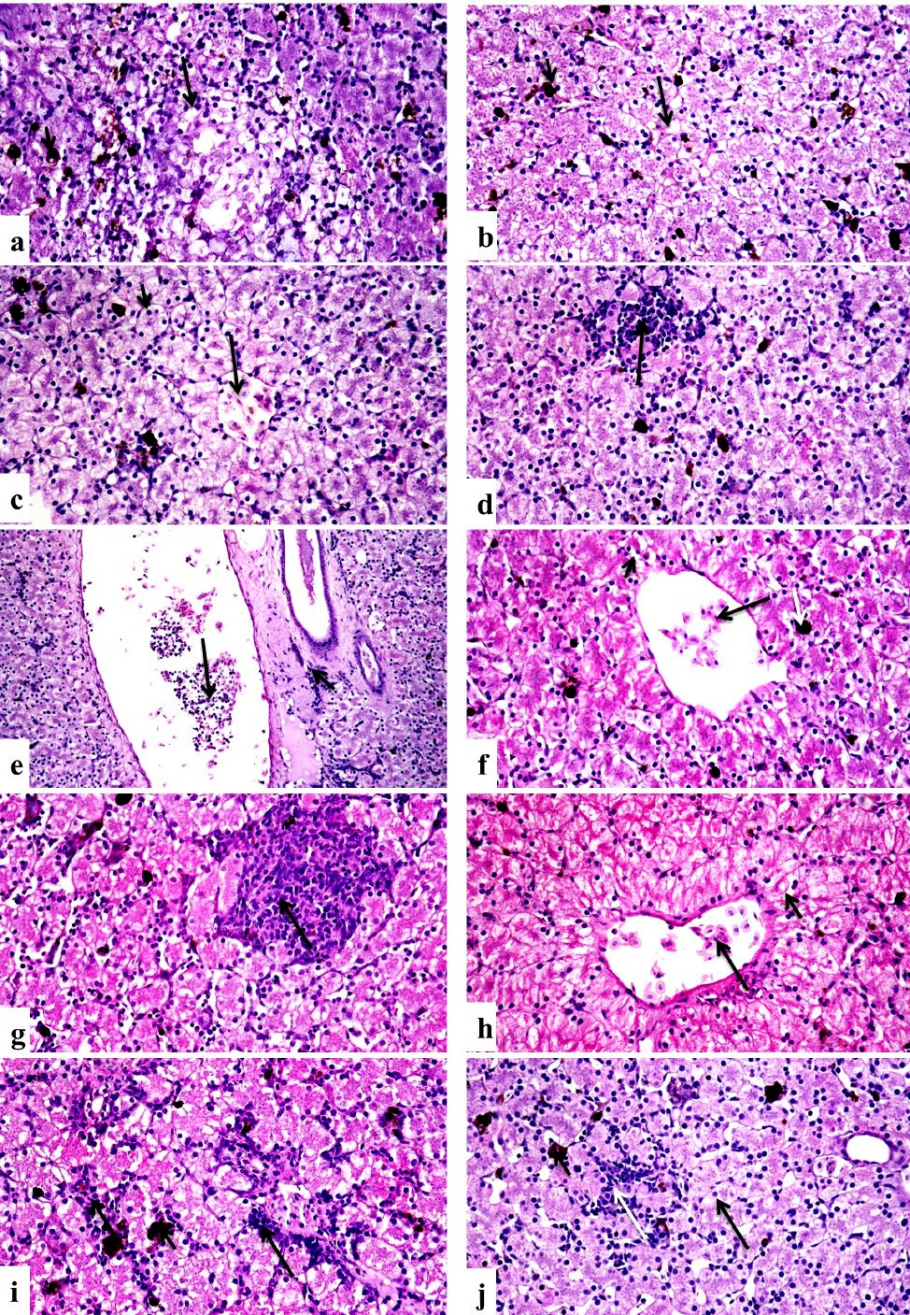
Photomicrographs of sections in the liver tissues of different groups of the African-Egyptian toad treated with different nanomaterials: (**a**) A section in the group treated with group B, showing vacuolar degeneration of hepatocytes (long arrow) and activation of melanomacrophages (short arrow). (**b**) A section in the group treated with group C, showing vacuolar degeneration of hepatocytes (long arrow) and activation of melano-macrophages (short arrow). (**c**) A section in the group treated with group C showing central vein congestion (long arrow) and hepato-cellular vacuolation (short arrow). (**d**) A section in the group treated with group D, showing focal area of mononuclear inflammatory cells infiltration (arrow). (**e**) A section in the group treated with group E showing portal area fibrosis and mononuclear inflammatory cells infiltration (short arrow), congestion of portal blood vessels(long arrow). (**f**) A section in the group treated with group F, showing mild hepatocellular degeneration (short arrow), mild congestion of central vein (long arrow) and activation of melano-macrophages (white arrow). (**g**) A section in the group treated with group F, showing mononuclear infiltration (arrow). (**h**) A section in the group treated with group G, showing moderate vacuolar degeneration of hepatocytes (short arrow) with central vein congestion (long arrow). (**i**) A section in the group treated with group G, showing focal areas of mononuclear inflammatory cells infiltration (long arrows) and activation of melano-macrophages (short arrow). (**j**) A section in the group treated with group H, showing mild hepatocellular vacuolation (long arrow), few mononuclear infiltration (white arrow) and activation of melanomacrophages (short arrow). (H & E X400)
2. Kidneys In the case of the control group, the kidneys displayed normal structure (Fig. 8a), whereas the group that had been exposed to group D had minor vacuolation of the renal tubular lining epithelium (Fig. 8b). The renal tubular lining epithelium of the group treated with group E, however, revealed vacuolation and necrosis (Fig. 8c). The renal tubular lining epithelium exhibited minor degradation in the group treated with group B. In contrast, only a few mononuclear inflammatory cells were infiltrated between the renal tubules (Fig. 8d). While the kidney tubular lining epithelium of the group exposed to group C showed significant vacuolar degeneration and necrosis (Fig. 8f) as well as mononuclear inflammatory cell infiltration between renal tubules (Fig. 8g). While the F group demonstrated minimal infiltration of mononuclear inflammatory cells and modest epithelial degradation of the renal tubular lining (Fig. 8h). While the kidney tubular lining epithelium of the group exposed to group G similarly exhibited minor vacuolar degeneration and a minimal infiltration of inflammatory cells (Fig. 8i). The histological structure of the renal tissues was practically normal in the group treated H; I (Fig. 8j).
  **Fig. 8:**
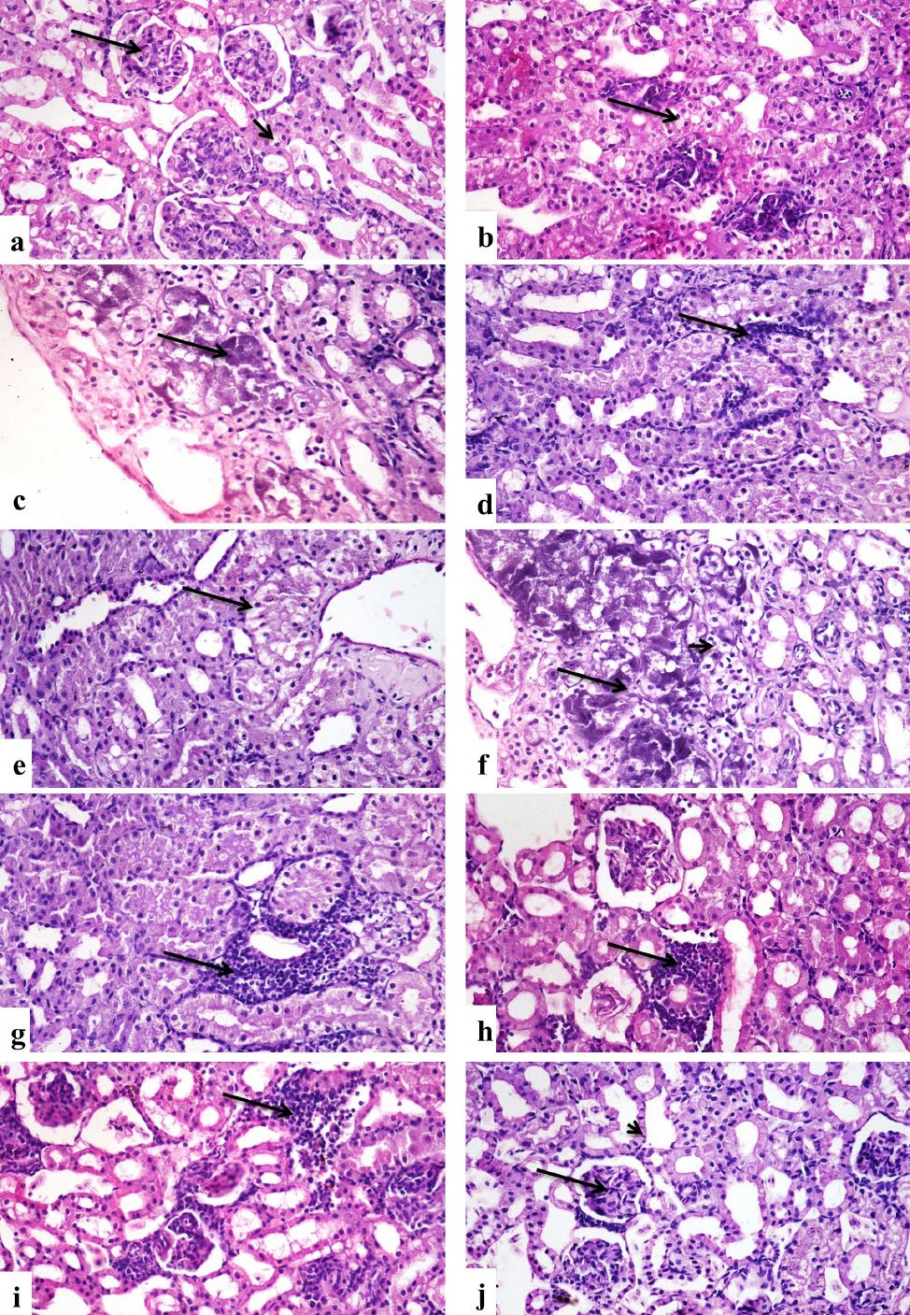
Photomicrographs of sections in the kidneys tissues of different groups of the African-Egyptian toad treated with different nanomaterials: (**a**) A section in the control group, showing normal histological structure of renal corpuscles (long arrow) and renal tubules (short arrow). (**b**) A section in the group treated with group D, showing mild vacuolar degeneration of renal tubular lining epithelium (arrow). (**c**) A section in the group treated with group E showing vacuolar degeneration and necrosis of renal tubular lining epithelium (arrow). (**d**) A section in the group treated with group B showing few mononuclear inflammatory cells infiltration (arrow). (**e**) A section in the group treated with group B showing mild degeneration of renal tubular lining epithelium (arrow). (**f**) A section in the group treated with group C showing severe vacuolar degeneration (short arrow) and necrosis of renal tubular lining epithelium (long arrow). (**g**) A section in the group treated with group C showing mononuclear inflammatory cells infiltration (arrow). (**h**) A section in the group treated with group F, showing few mononuclear inflammatory cells infiltration (arrow). (**i**) A section in the group treated with group G, showing few mononuclear inflammatory cells infiltration (arrow). (**j**) A section in the group treated with group H, showing normal histological structure of renal corpuscles (long arrow) and renal tubules (short arrow). (H & E X400)

**Histopathological lesion scoring**: the recorded lesions in kidney were evaluated according to severity as illustrated in Table 8.

**Table 8:** Scoring of histopathological alterations in liver and kidneys of all experimental toad groups

Organs	Lesions	G. A	G.B	G.C	G.D	G.E	G.F	G.G	G.H
Liver	Vacuolar degeneration of hepatocytes.	0	1	3	2	3	1	2	1
	Hepatocellular necrosis.	0	0	3	1	2	0	0	0
	Congestion of central vein.	0	1	2	2	2	1	1	1
	Activation of melanomacrophages.	0	1	2	2	3	1	1	2
	Mononuclear inflammatory cells infiltration.	0	1	1	1	2	1	1	1
	Portal fibrosis.	0	1	2	0	3	0	0	0
Kidneys	Interstitial infiltration of mononuclear cells.	0	0	0	1	2	1	2	0
	Degeneration of tubular lining epithelium.	0	1	2	1	3	1	2	0
	Necrosis of tubular lining epithelium.	0	0	2	0	3	0	0	0

The scoring system was designed as: score 0 = absence of the lesion in all the groups of the tested toads. Each group (*n* = 3), score 1= (< 30%), score 2= (< 30 – 50%), score 3= (> 50%)

## Discussion

In the current study rat group treated with chitosan nanoparticles B showed mild interstitial nephritis, vacuolation or necrosis of few tubular lining epithelium. While rat group treated with chitosan nanoparticles C revealed vacuolation and thickening in glomerular tuft, interstitial tissue was heavily infiltrated with mononuclear inflammatory cells, vacuolation and necrosis of most tubular lining epithelium with congestion of interstitial blood vessels. Chitosan is a relatively safe substance due to biodegradable and biocompatible properties; however, several studies confirmed the limited cytotoxicity of chitosan nanoparticles [37–39]. Further research work is required to investigate toxicity of chitosan nanoparticles on humans and other organisms [40]. Green and environmentally safe synthesis of chitosan derivatives should be developed to protect our environment as they are considered a promising material for biomedical applications [41]. Chitosan nanoparticle damages the plasma membrane of cells leading to LDH release followed by mitochondrial injury and production of Reactive Oxygen Species (ROS). ROS-mediated cell necrosis is a key pathogenesis in the injury induced by chitosan nanoparticles [42]. In the current study, group treated with silver nanoparticles D showed mild interstitial nephritis with vacuolation of tubular lining epithelium and glomerular congestion. While, rat group treated with silver nanoparticles E showed capsular thickening with edema and inflammatory cells, interstitial nephritis, glomerular and interstitial blood vessels congestion with vacuolation and necrosis of tubular lining epithelium. These abnormalities were attributed to formation of ROS by silver nanoparticles, that interact with and damage both proteins and DNA. Silver nanoparticle can induce apoptosis and necrosis in plant and animal cells [43]. Similar results were also obtained when silver nanoparticles was tested on zebra fish [44]. Group treated with chitosan-silver F nanocomposite revealed cystic dilatation and necrosis of a few number of renal tubules with mild interstitial nephritis, some renal tubules showed renal casts inside their lumen. Whereas group treated with chitosan-silver nanocomposite (G) showed congestion of glomerular tuft and interstitial blood vessels with renal tubular casts and few inflammatory cells infiltration and vacuolation or necrosis in some renal tubular epithelium. Chitosan-silver nanocomposite have antibacterial [45]; antioxidant [46] properties, which can be used in food applications. They were also used as insecticides against mosquito larvae (Shamseldean et al. [20]. , ) and bedbugs [16]. While chitosan-silver nanocomposites have all these positive characters, they also can cause adverse biological effect, thus, there is a great concern about health and environmental risks related to their use. In addition, some studies proved that toxicity of chitosan-silver nanocomposite was dose-dependent [47]. In contrast, lavender oil nanoemulsion was used as a strong repellant against vector insects such as mosquitoes [48], and as a mosquito larvicide [20]. In the present work impact of H and I groups of lavender nanoemulsion on kidneys of non-target animals was mild showing interstitial nephritis and vacuolation of a few tubular lining epithelium. The research work by Mekonnen et al. [49]. , in a toxicity study as the basis of safety assessment also proved that lavender nanoemulsion had no toxic effects on kidneys with no histopathological changes. In addition, the immunostaining expression in the groups F and G chitosan-silver and/or lavender nanoemulsion ranged from zero to a very weak positive immunological response. The expression of TNF-α, an inflammatory cytokine produced by macrophages during acute inflammation, and BAX, a pro-apoptotic member of the Bcl-2 family, suggested that the toxic effects of nanoparticles are produced to varying degrees by oxidative stress and apoptosis depending on the type and dose of the used nanoparticles [43]. In contrast, rapid IL-6 synthesis which is a powerful acute phase response inducer aids in host defense during infection and tissue damage, however overproduction of IL-6 is a factor in a disease pathology. When pathogens are identified by toll-like receptors (TLRs) at the site of infection or tissue damage, myeloid cells, such as macrophages and dendritic cells, produce the chemical during the innate immune response necessary for the development of B-cells into immunoglobulin-secreting cells during the adaptive immunological response. It has a significant impact on CD4 + T cell subgroup differentiation. A crucial component for the growth of T follicular helper (Tfh) cells, that are necessary.

The only inflammatory cytokine that may be detected in considerable amounts in the blood during fever is interleukin-6; Cartmell et al. [50]. ; In both experimental animals and people, a rise in circulation and cerebrospinal fluid (CSF) IL­6 concentrations is closely associated with the onset of fever and toxicity. Locally at the inflammatory site, IL-1β and IL-6 are produced in response to LPS injection. The circulatory system can be exposed to locally generated IL6, which appears to be a crucial mediator of the febrile reaction to local LPS-induced inflammation. When injected into a subcutaneous air pouch, IL­6 alone had no effect on body temperature; however, when combined with a non­pyrogenic dose of IL­1β, IL­6 significantly raised body temperature and when combined with a pyrogenic dose of IL­1β, which exacerbated the febrile response. Therefore, it appears that IL6 works in conjunction with IL1β and maybe other soluble mediators to either cause or worsen the fever; Cartmell et al. [50, 51].

The immune systems of teleost fish are not entirely the same as those of other vertebrates, such mammals. They consist of the digestive tract, kidney, liver, spleen, and thymus.

In reaction to environmental stimuli, fish can had an immune response and change the patterns of gene expression. Heavy metals, which serve as a defense against numerous pests and stressors, are frequently found and poisonous in the fish spleen [52–54].

Similar to other vertebrates, the spleen of fish serves as the primary filter for blood-borne antigens as well as any external stimuli and is also involved in immunopoietic processes. It is involved in hematopoiesis in teleost fish and may provide immunological roles similar to those of mammalian lymph nodes (fish do not have lymph nodes). It is smooth, dark crimson in colour, and located in the lower posterior abdominal cavity. The outer capsule of the spleen is made of pulp matrix and connective tissue. Both lymphopoietic white pulp and hematopoietic red pulp are present in the pulp. The spleen is the primary location of thrombocyte formation and may hold a significant amount of mature erythrocytes that can be discharged into the bloodstream as necessary.

## Conclusion

Green nanoemulsion of the lavender essential oil was relatively more effective, and safe, which could be used as insecticides against mosquito larvae than the metal-based nanomaterials which is based upon the histopathological lesion on rats kidney and liver and kidney of toad.

## Data Availability

No datasets were generated or analysed during the current study.

## Abbreviations

AgNP: silver nanoparticle
ALT: alanine aminotransferase
AST: aspartate aminotransferase
IL: interleukin
ROS: reactive oxygen species
